# Supplementary material for: Transcription-coupled eviction of histones H2A/H2B governs V(D)J recombination
Source: EMBO J. 2013 Mar 5;32(10):1381–92. doi: 10.1038/emboj.2013.42 (PMC3655464; doi:10.1038/emboj.2013.42)
Supplement: Supplementary Information [file emboj201342s1.pdf]

Bevington Supplementary Figure S1

A

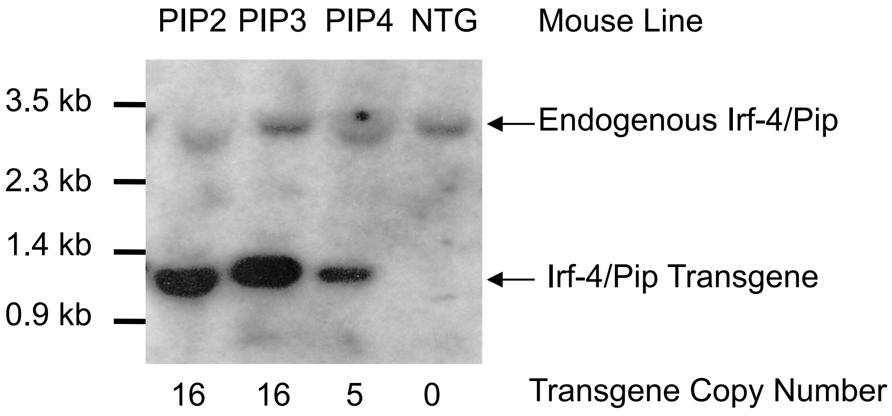

B

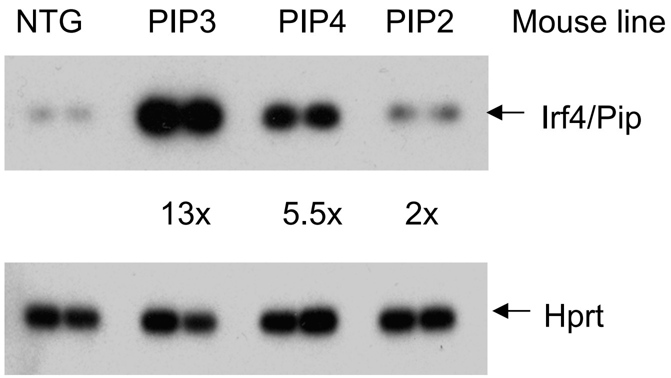

Bevington Supplementary Figure S2

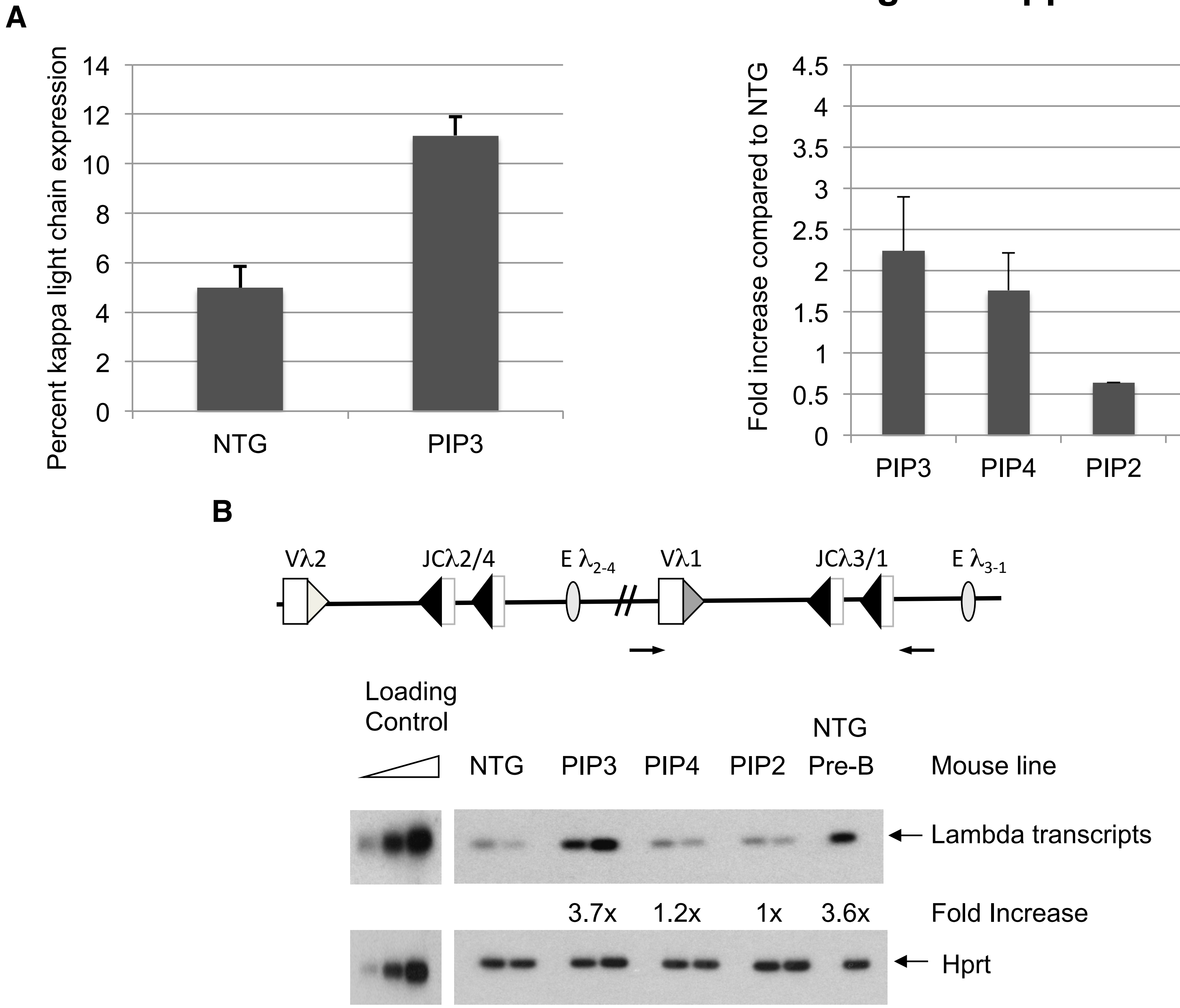

Bevington Supplementary Figure S3

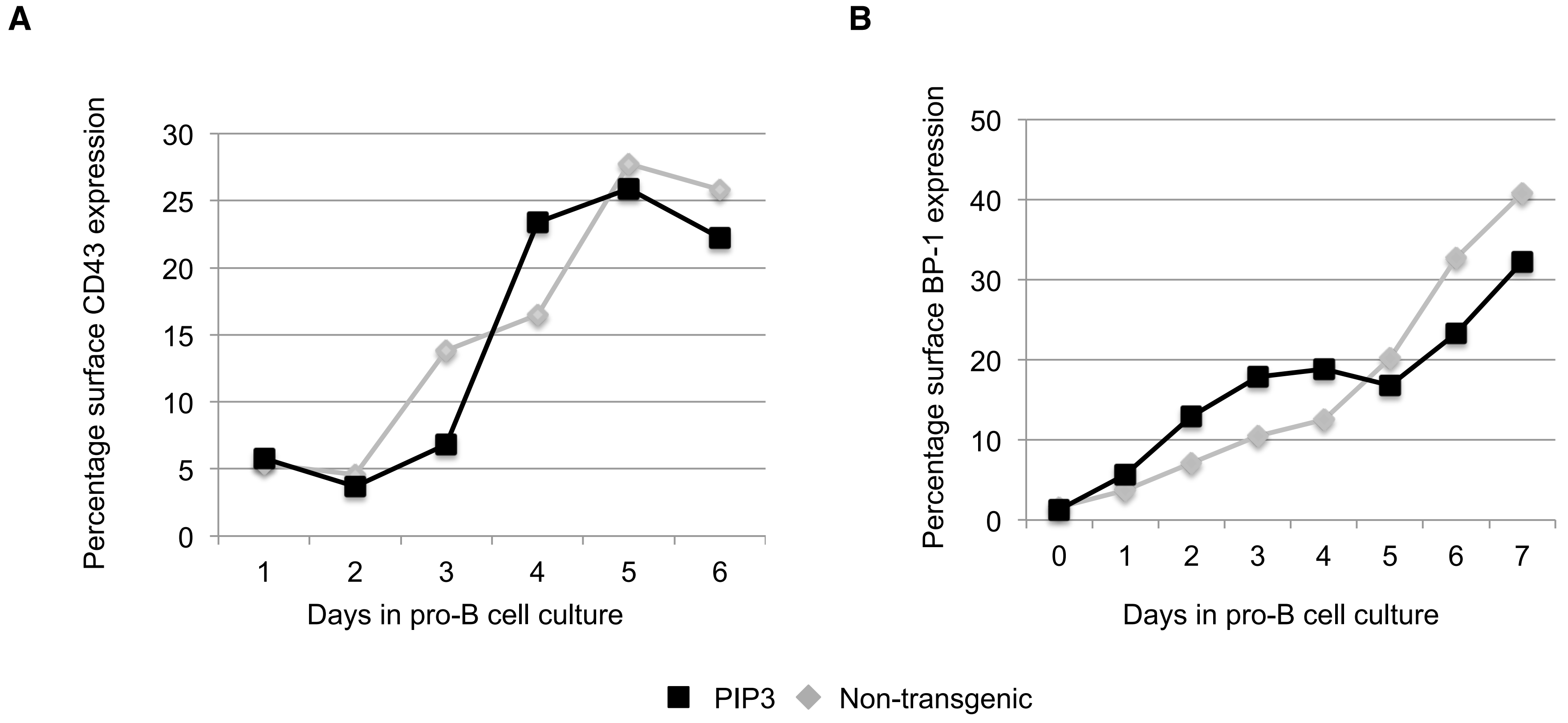

Bevington Supplementary Figure S4

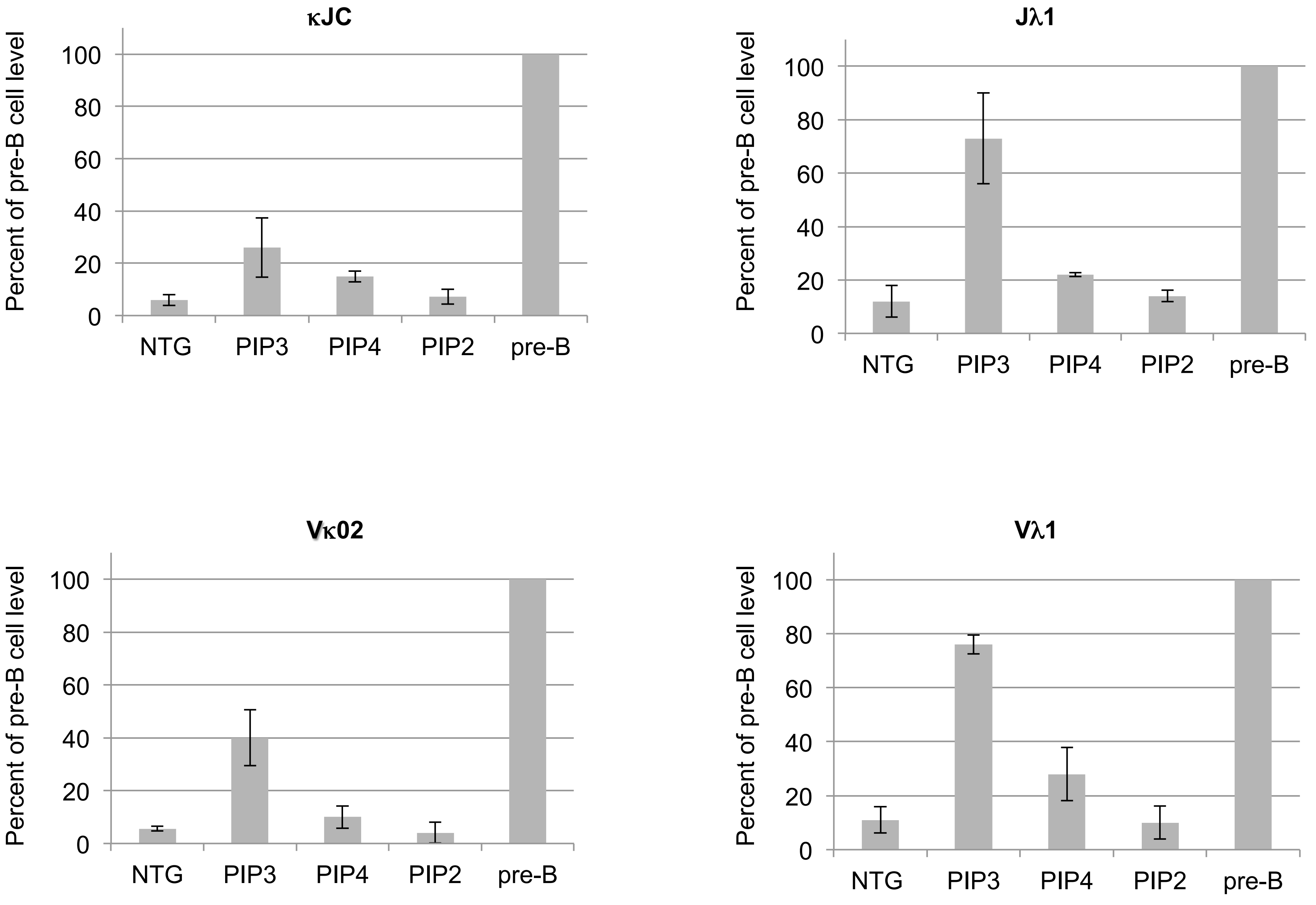

Bevington Supplementary Figure S5

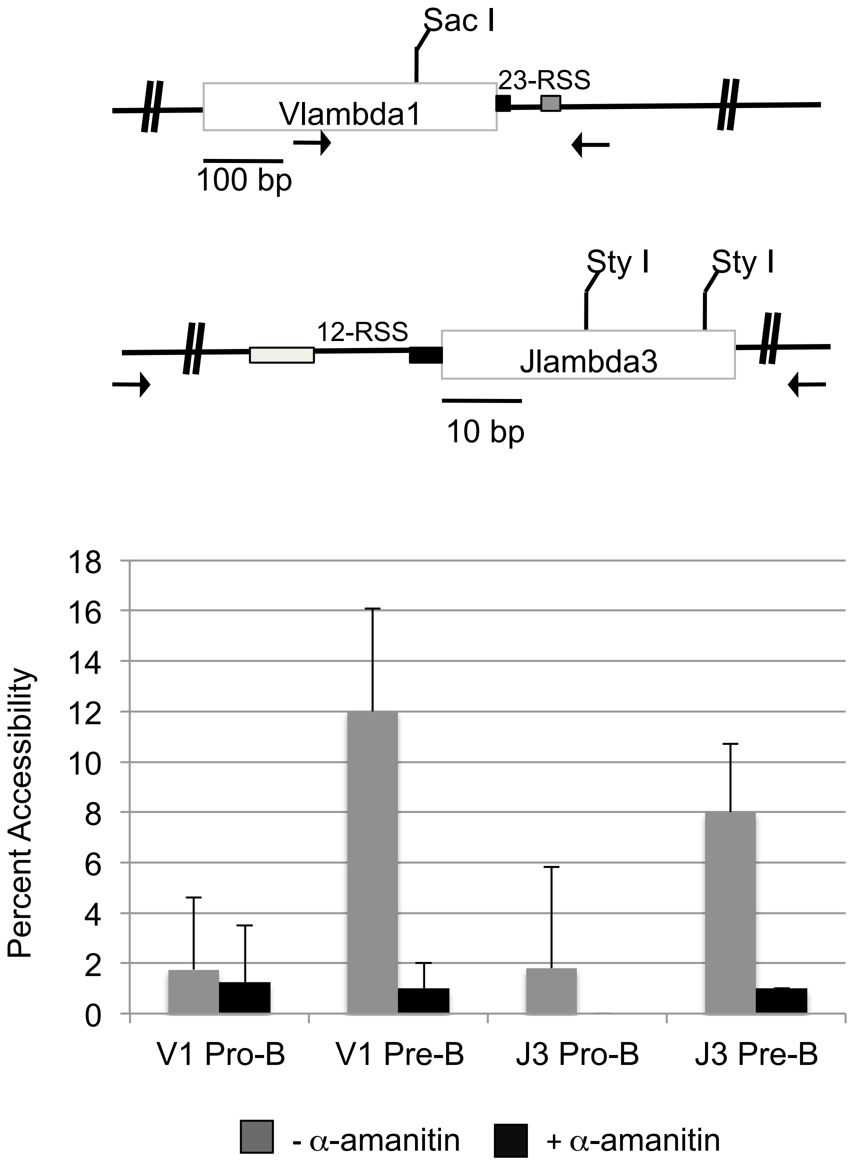

Bevington Supplementary Figure S6

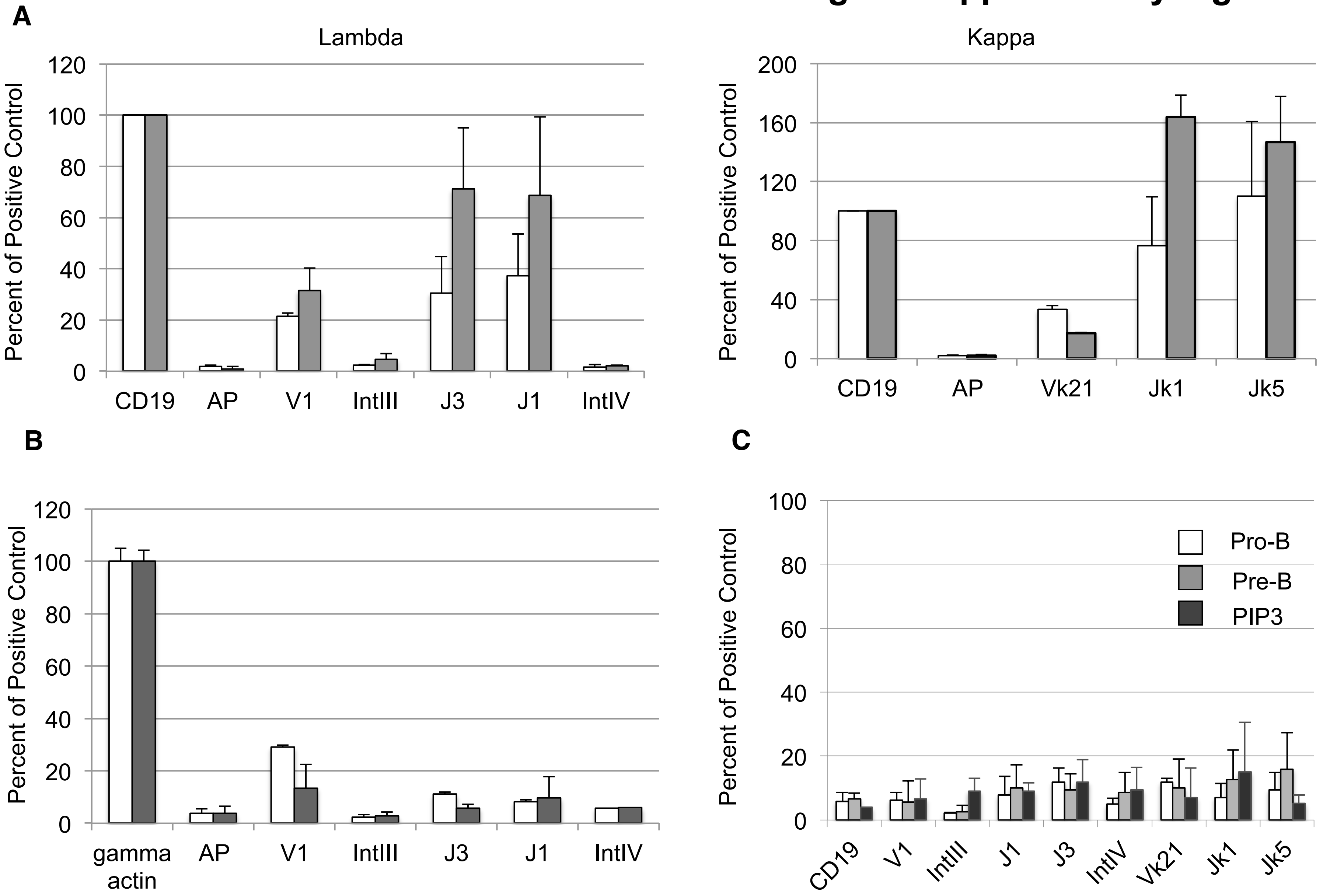

Bevington Supplementary Figure S7

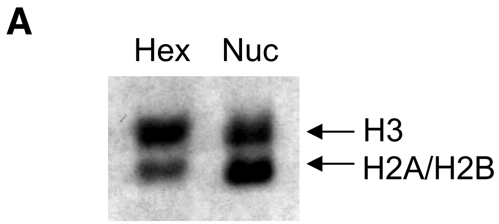

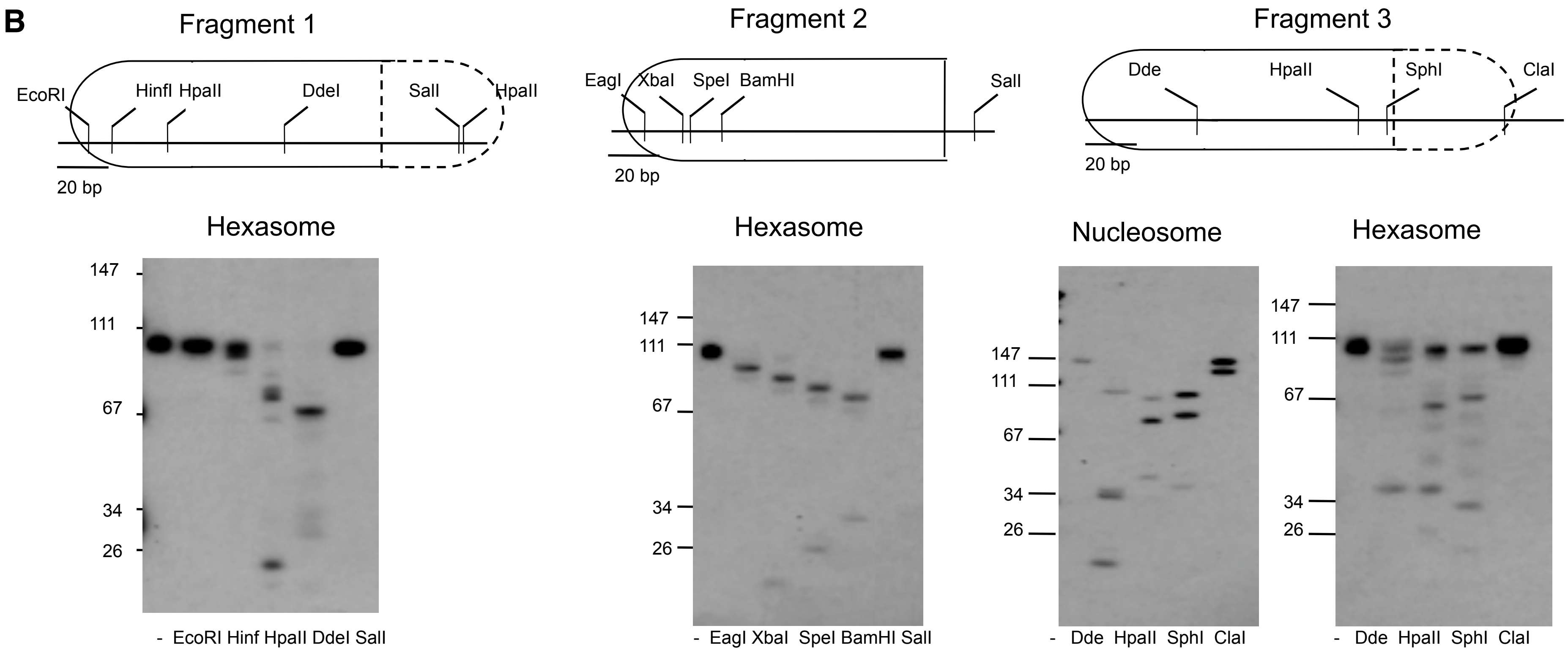

Bevington Supplementary Figure S8

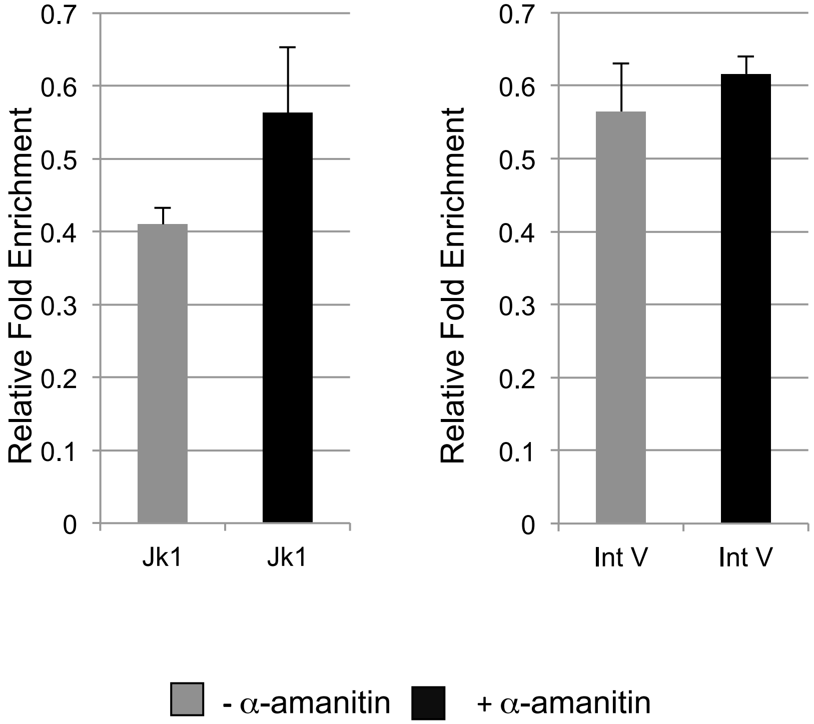

Supplementary Figure Legends and Tables

Supplementary Figure S1: Copy number of transgenic lines and *Irf4* mRNA levels. A) Southern blot to determine the copy number of the transgenic lines (PIP2, 3, 4); DNA from a non-transgenic mouse (NTG) is included as a control. The copy number was calculated by dividing the intensity of the transgene band by that of the endogenous band, correcting for the lengths of the respective bands. **B)** Levels of *Irf4* mRNA as determined by semi-quantitative PCR plus Southern blotting. The mRNA levels were normalised to the level of *Hprt* transcripts. Duplicate samples from each mouse line are shown.

Supplementary Figure S2: Premature recombination of light chains in pro-B cells correlates with their increased transcription and translation. A) Left: Primary pro-B cells, which had been expanded in culture for 7 days, were analysed for the percent cell surface expression of *Igκ* by flow cytometry. Cells were analysed from non-transgenic mice (NTG) and from the PIP3 transgenic line. The quantification of the average level of expression from three different experiments is shown with the standard deviation. Right: Levels of *Vκ21-Jκ1* recombination analysed by qPCR using DNA derived from primary pro-B cells from the transgenic lines indicated. Recombination was determined using the primers Vk21F and Jk1RSSR and the amounts of DNA were normalised to an intergenic region. Standard deviation from the average of three experiments is shown. **B)** Southern blot detecting rearranged *Igλ* light chain transcripts. The levels of transcripts were measured in pro-B cells using two samples from each of the transgenic lines (PIP2, PIP3 and PIP4) and non-transgenic mice (NTG). mRNA levels were normalised using the level of *Hprt* transcripts. The primers used to amplify the rearranged lambda transcripts are indicated by arrows in the schematic. The fold increase shown is compared to pro-B cells from non-transgenic mice.

Supplementary Figure S3: The levels of pro-B cell markers in the non-transgenic and PIP3 transgenic mice. The levels of CD43 **(A)** and BP1 **(B)** were measured by flow cytometry each day for 7 days after the primary pro-B cells were put into culture. Since these cell surface markers change equivalently between cells from transgenic and

non-transgenic mice, this suggests that increased levels of IRF4 do not alter the kinetics of B cell development and that the differences in *Igκ* and *Igλ* recombination are not due to changes in the time the cells spend in the pro-B cell compartment.

Supplementary Figure S4: **The levels of non-coding transcripts at the IgL loci in pro-B cells from the different transgenic lines.** The levels of non-coding transcripts were determined by PCR plus Southern blotting as shown in Figure 3. The data were quantified using a phosphorimager and standard deviation from the average of three experiments is shown. The levels relative to those in pre-B cells are shown in each case.

Supplementary Figure S5: Accessibility at the Igλ locus RSSs. The level of accessibility at the $V\lambda_1$ and $J\lambda_3$ gene segments increases in pre-B cells. Upper: Schematics of the $V\lambda_1$ and $J\lambda_3$ gene segments and RSSs, showing the restriction sites used to probe accessibility. Labels are as for Figure 5A. Lower: Accessibility was measured in pro-B cells and pre-B cells that had been purified by flow cytometry and cultured in the absence (grey bars) or presence (black bars) of α-amanitin.

Supplementary Figure S6: Histone H3 acetylation and H3K36me3 at the *Igκ* and *Igλ* loci and control IgG chromatin immunoprecipitation. A) Histone H3 acetylation levels at the *Igλ* locus (left) and *Igκ* locus (right) in primary pro-B and pre-B cells from non-transgenic mice. **B)** Histone H3K36me3 levels at the *Igλ* locus. The level of enrichment is shown as a percentage of H3K36me3 at the *γ-actin* gene. **C)** Control immunoprecipitations were carried out using anti-IgG antibody. The level of enrichment is shown as the percentage of the enrichment of the level of H3 acetylation at the CD19 gene. Labels are as for Figure 4.

Supplementary Figure S7: Characterisation of nucleosomes and hexasomes. A) The stoichiometry of histones H2A and H2B is reduced in the hexasome compared to the nucleosome. Silver stained gel that had been loaded with similar amounts of nucleosome and hexasome reconstituted onto Fragment 3. Recombinant histones H2A

and H2B have a very similar molecular weight and co-migrate. The bands for histone H3 and H2A/H2B are shown; histone H4 is not shown since it stained weakly, most likely due to its co-migration with the DNA fragment from this reconstitute. **B)** Mapping of the hexasome and nucleosome positions by micrococcal nuclease. The upper panel shows the restriction map of each fragment together with the most favoured positions of the nucleosomes and hexasomes, as determined by the mapping experiment below. The lower panels show the mapping experiment: Reconstitutes were digested with micrococcal nuclease and the DNA protected was gel purified. Following end-labelling, it was digested with the restriction enzymes shown beneath the gels. From this digestion pattern, the position of the nucleosome or hexasome was calculated.

Supplementary Figure S8: H2A is depleted at the *Jκ1* RSS in a transcription-dependent manner. Left: Level of H2A at the *Jκ1* RSS in the presence and absence of α-amanitin. Right: Relative levels of H2A at the Intgene V region that is not known to be transcribed. Levels were normalised to a second non-transcribed region (Int III). The standard deviation from the average of three experiments is shown.

Supplementary Table S1

Primers used to characterise the transgenic mice

Primers	Sequence	Annealing temperature/ Cycles/Ext time
**mRNA**		
IRF4 expressionF	GGAGCAAAGCAGCTCACTTTG	56°C/25x/30s
IRF4 expressionR	CATGGGGTGGCATCATGTAG	
HPRTF	GGGGGCTATAAGTTCTTTGC	58°C/23x/30s
HPRTR	TCCAACACTTCGAGAGGTCC	
**Recombination**		
Lambda-recF	ATTCTCAGGCTCCCTGATTTGGAGACAAGG	54°C/26x/30s
Lambda-recR	CAAAAAGCACCTCAAGTCTTGG	
Vk	GGCTGCAGSTTCAGTGGRTCNGGRAC	60°C/26x/30s
Jk1SouthR	GCCACAGACATAGACAACGGAAGAAAG	
Jk5	AGGTTGCCAGGAATGGCTCA	
B-globinF	TGGGCAAATACCAGACAC	56°C/22x/30s
B-globinR	CACATCTTCCAAAGCAG	
**Non-coding Transcripts**		
Vlambda1/2F	TGAATTATGGCCTGGATTTCA	54°C/26x/30s
Vlambda1/2R	GCCACCTGTTAAGAAGATGGTAGTTA	
lambdaJCF	GTCTCCTCAAGCTGTCACTGG	56°C/26x/30s
lambdaJCR	ACCTAGGAACAGTCAGCACG	
kappaJCF	GAGGGGGTTAAGCTTTCGCCTACCCAC	60°C/24x/30s
kappaJCR	GTTATGTCGTTCATACTCGTTCCTTGGTCAAC	
STVk02F	TGATGAGTCCTGCCCAGTTCCTG	60°C/23x/30s
STVk02R	CTCCCAAATCCTCAGCCTCCACTC	
**Rearranged Transcripts**		
LambdaTF	GGCACAGACTGAGGATGAG	54°C/25x/30s
LambdaTR	CAGTCAGTTTGGTTCCTCCA	

Supplementary Table S2

Primers used for Chromatin Immunoprecipitation and RSS accessibility

**Primers**	Sequence	Annealing °C
CD19F	GAGGGGGACAAGTGTTGTGGATTT	57
CD19R	TGAACCATGGGTGTCTGTGAGGG	
APF	GCTTTCCCTCATTGGGTTCTG	55
APR	ACTCGACAGGTGGACAATAGCA	

IRF4prF	CCCAACCTGGCCATGAGCAG	60
IRF4prR	CCTGGCTTTCTTCAGCTCCTCC	
GapdhF	ACTTTCTTGTGCAGTGCCAGC	56
GapdhChIPR	GCACACTTCGCACCAGCATC	
**Lambda Locus**		
IntIIIF	CAAGGAAAGGCCAACCAATA	54
IntIIIR	TAACCCTTTCCCCAGCTCTT	
IntIVF	AAAGTCCTTTTGCTCCTCA	54
IntIVR	ACACAAGGTAGGGCCAGATG	
J3RSSF	TGCAGCTGTGAGAGAACAGG	55
J3RSSR	AGGACAGTGACCTTGGTTCC	
J1RSSF	ACCAGGAGCTGCATACATCA	55
J1RSSR	CAGTCAGTTTGGTTCCTCCA	
V1RSSF	GGCACAGACTGAGGATGAG	54
V1RSSR	GGGAGATGTAGCCACCTGTT	
El3-1F	GACATTACAAGCTCTGTGGAGG	56
El3-1R	GCTAATGGACTTGGTTTCAGTTCC	
IntVF	CTCCCTGGGCATGTCAC	56
IntVR	CCGCTCCTCACATCTCCC	
**Kappa Locus**		
VK4F	CAGCACTGAAGTACAGTGG	54
VK4R	GAAAATAAACTGCAGCCAGTGG	
VK24F	GGAGGCTGAGGATGTGGG	54
VK24R	CATCATGATACATCCAGACAAGG	
VK21F	ATCCTGTGGAGGCTGATGAT	54
VK21R	GCAACCCTAGGAGGTTTTTG	
JK1RSSF	GGGTTTTTGTACAGCCAGACA	55
JK1RSSR	CAACGGAAGAAAGAGACTTTGG	
JK5RSSF	GCTGGGCTTTTCCTCTGAATT	55
JK5RSSR	CTATGACATGCCCTCTCTACAAA	
IntgeneF	GACAGTGTTTCTCTGTATAGCC	54
IntgeneR	GCAGTAGCTCTCCATTTTTAGC	
k3'EF	ACTGGCCTGAGATTAAAAAAAGTAATG	56
k3'ER	CAGTGTGACGGTAGCTATGACAGTTG	
Jk1StyF	CCGTTGTCTATGTCTGTGGCTTCTATG	58
Jk1StyR	CCAGAGAACATGTCTAGCCTATTCCC	
**Accessibility Primers**		
J1StyIF	ACCAGGAGCTGCATACATCA	55
J1StyIR	GCACCTCAAGTCTTGGAGAG	
J3StyIF	TGCAGCTGTGAGAGAACAGG	55
J3StyIR	AGGACAGTGACCTTGGTTCC	
StyIcF	CTCCCTGGGCATGTCAC	55
StyIcR	CCGCTCCTCACATCTCCC	
V1SacIF	GGCACAGACTGAGGATGAG	55
V1SacIR	GGGAGATGTAGCCACCTGTT	
SacIcF	GTCTGCAACCCCATAAGTGG	55
SacIcR	GTTTGTAAGACCAAGGGGCTTC	
Jκ3StyIF	GGAAGAGGGATAATTGTCTACCTAGGG	59
Jκ3StyIR	CCACAAACTCATACAAAGGACACAATGGG	
