# Supplementary material for: Transcription-coupled eviction of histones H2A/H2B governs V(D)J recombination
Source: EMBO J. 2013 Mar 5;32(10):1381–92. doi: 10.1038/emboj.2013.42 (PMC3655464; doi:10.1038/emboj.2013.42)

Explanation of source data

Figures 2A and 2B
The level of recombination in pro- and pre-B cells from non-transgenic bone marrow was measured at the same time as that in pro-B cells from the transgenic mice but the non-transgenic bone marrow samples were run on a separate gel. Importantly, however, this second gel had some of the same samples as on the first gel, allowing the exposures to be matched. Moreover, all lanes were quantified using a phosphorimager and normalised to a β-globin control PCR to further verify the comparison between gels. Originally, we spliced the lanes showing recombination in the pro- and pre-B samples onto the gel showing recombination in the transgenic mice. Since the pro- and pre-B non-transgenic samples were run on separate gels from the transgenic samples shown (albeit with internal controls for exposure) we have now separated these lanes in the revised Figure.

Annotated Source Data

Figures 2A and 2B

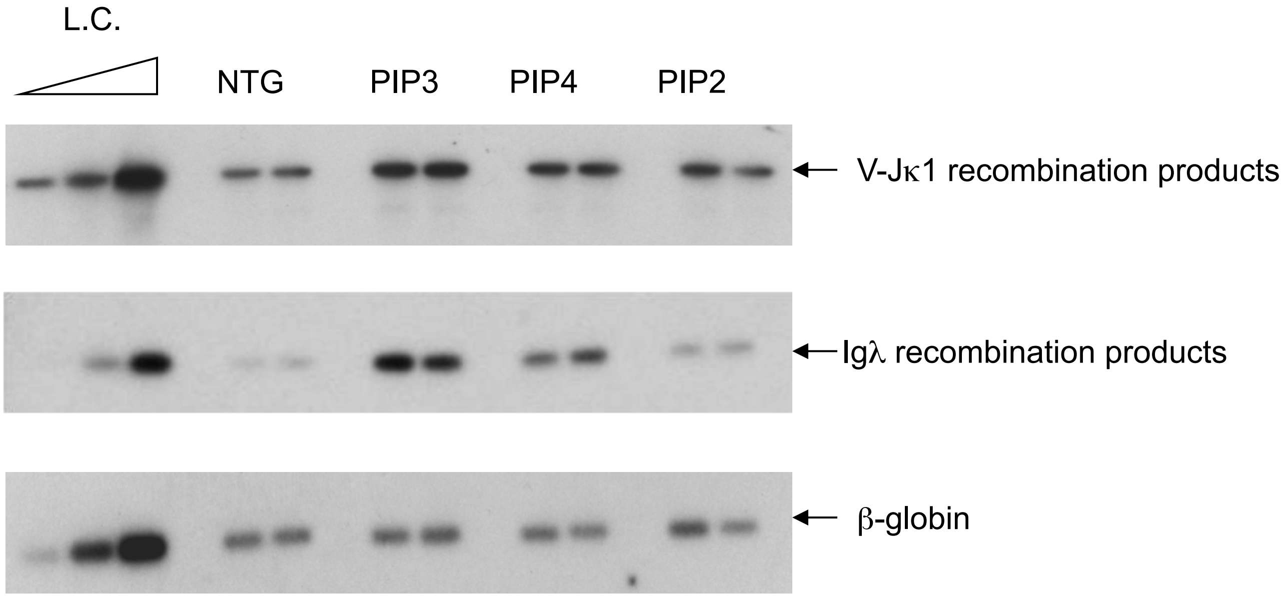

Scans of the original gels showing the level of recombination in the different PIP transgenic lines. These data are shown in Figures 2A and 2B.

Figure 2B

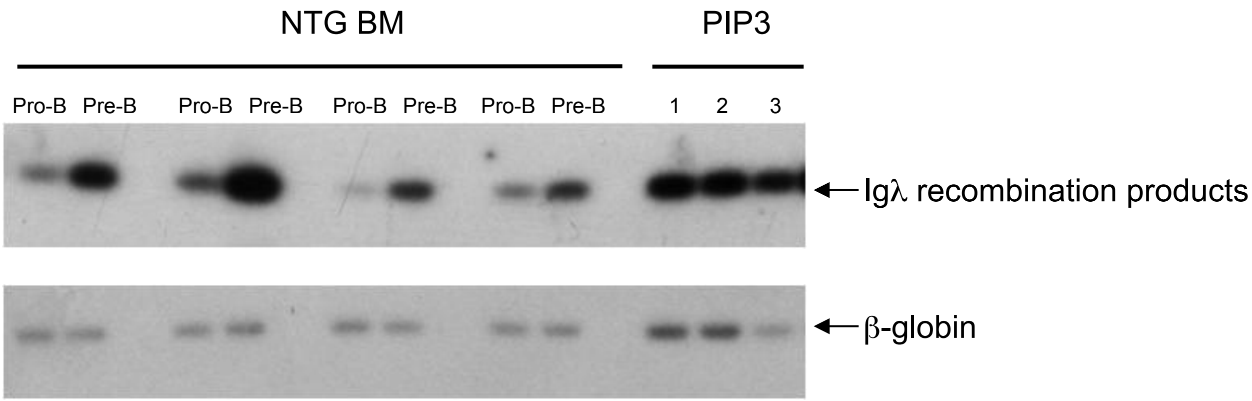

The lanes showing recombination in NTG BM on the far left of this dataset were used in Figure 2B.
The same PIP3 samples as shown in Figure 2B were also run on this gel (right) and used to normalise exposure levels.
All samples were normalised to β-globin as shown in the lower panel.

Figure 2A

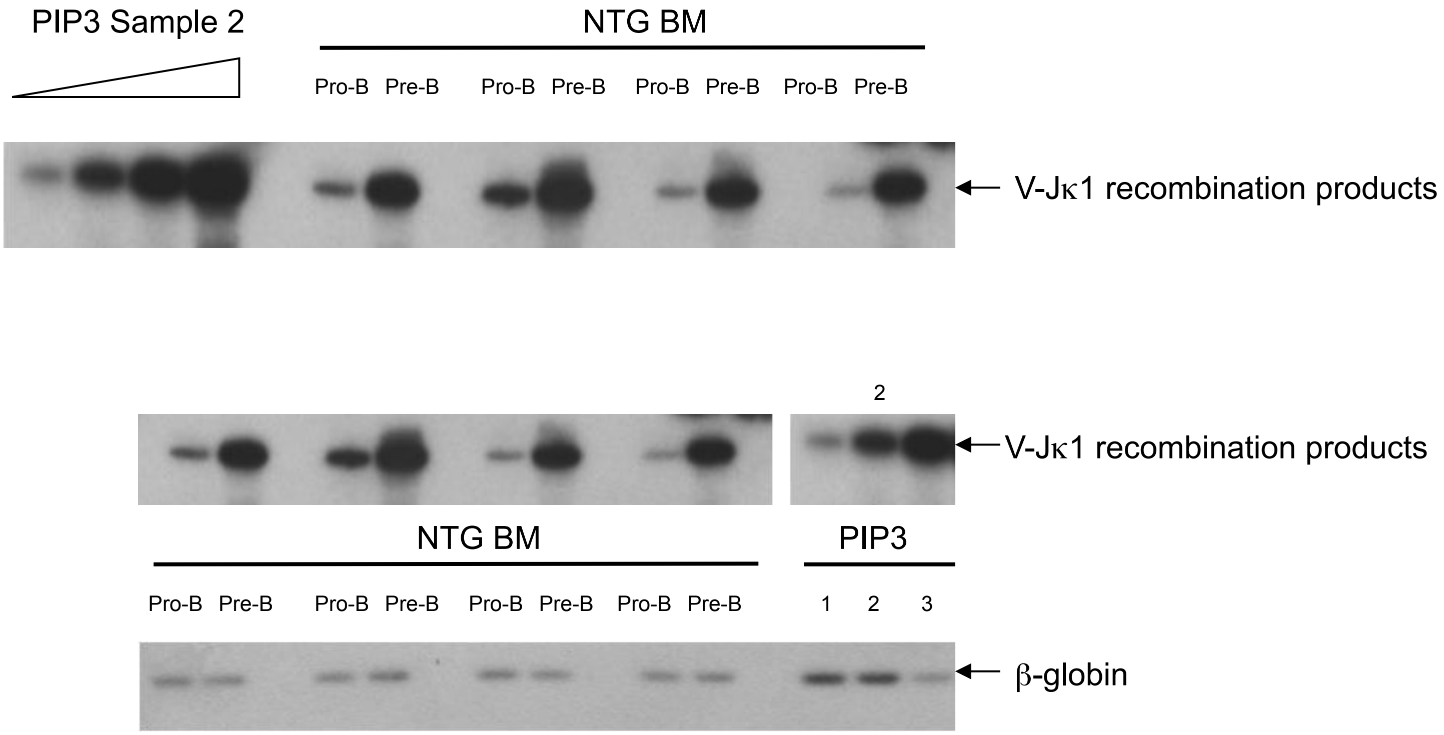

Upper panel: scan of original gel
Middle panel: PIP3 samples moved to right side of figure to match the corresponding β-globin loading controls (lower panel)
The lanes from the far left of this gel, showing the level of recombination in NTG pro- and pre-B cells, were used in Figure 2A
Sample 2 of the PIP3 loading control is the same as that shown in Figure 2A and was was used as a control for the exposure level

Scans of original gels

Recombination in the different PIP transgenic lines. These data are shown in Figures 2A and 2B

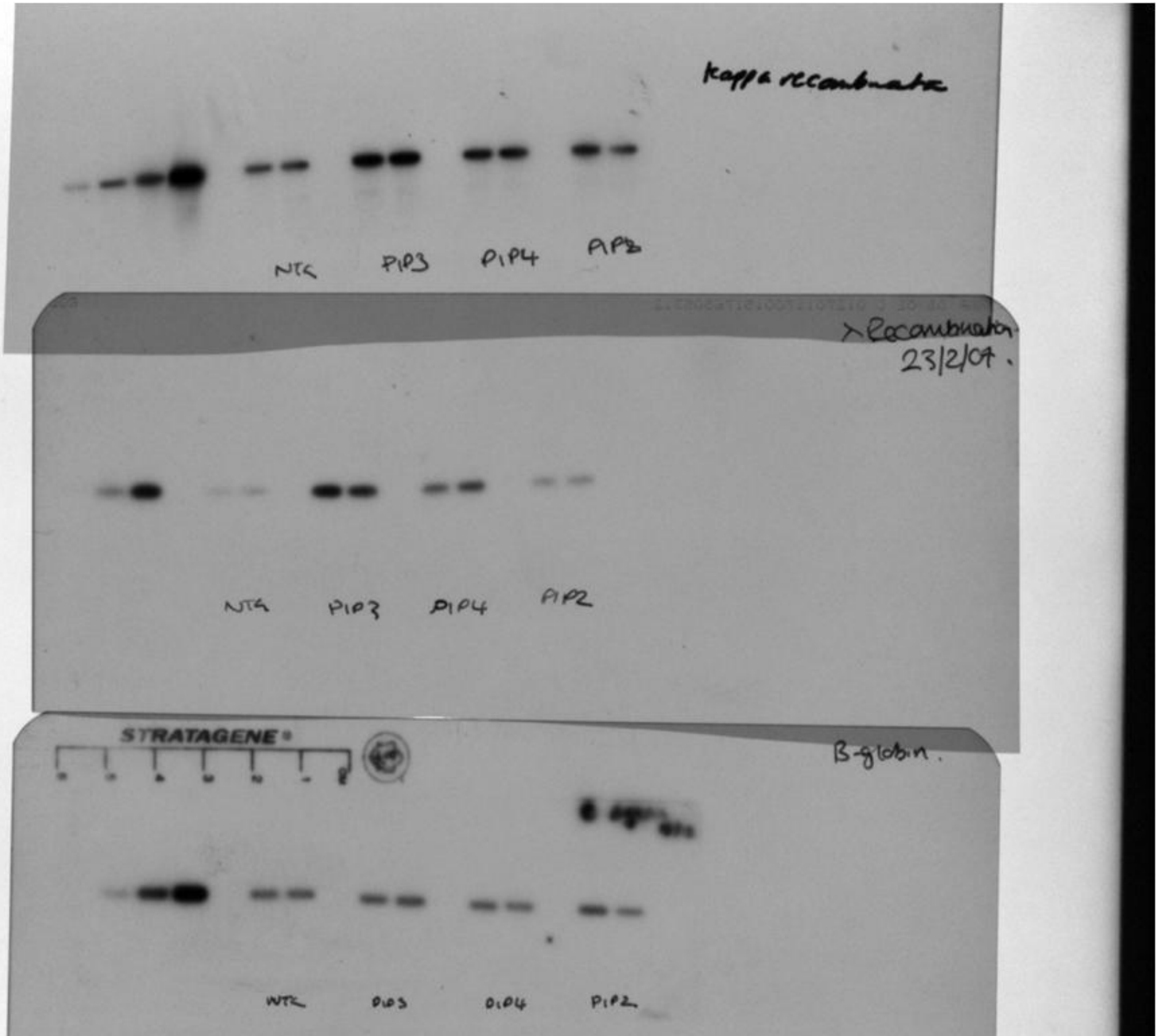

Recombination of Igλ in non-transgenic bone marrow pro- and pre-B cells

These data are shown in Figure 2B

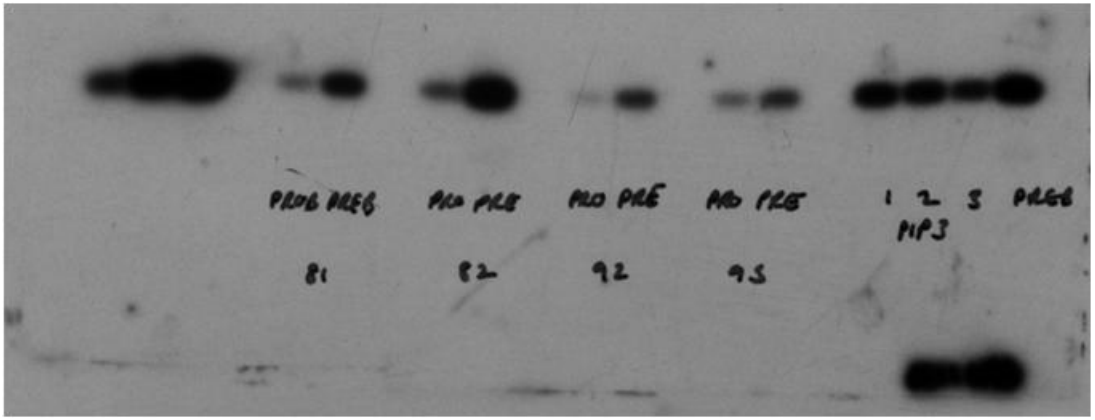

V-Jκ1 recombination in non-transgenic bone marrow pro- and pre-B cells.

These data are shown in Figure 2A

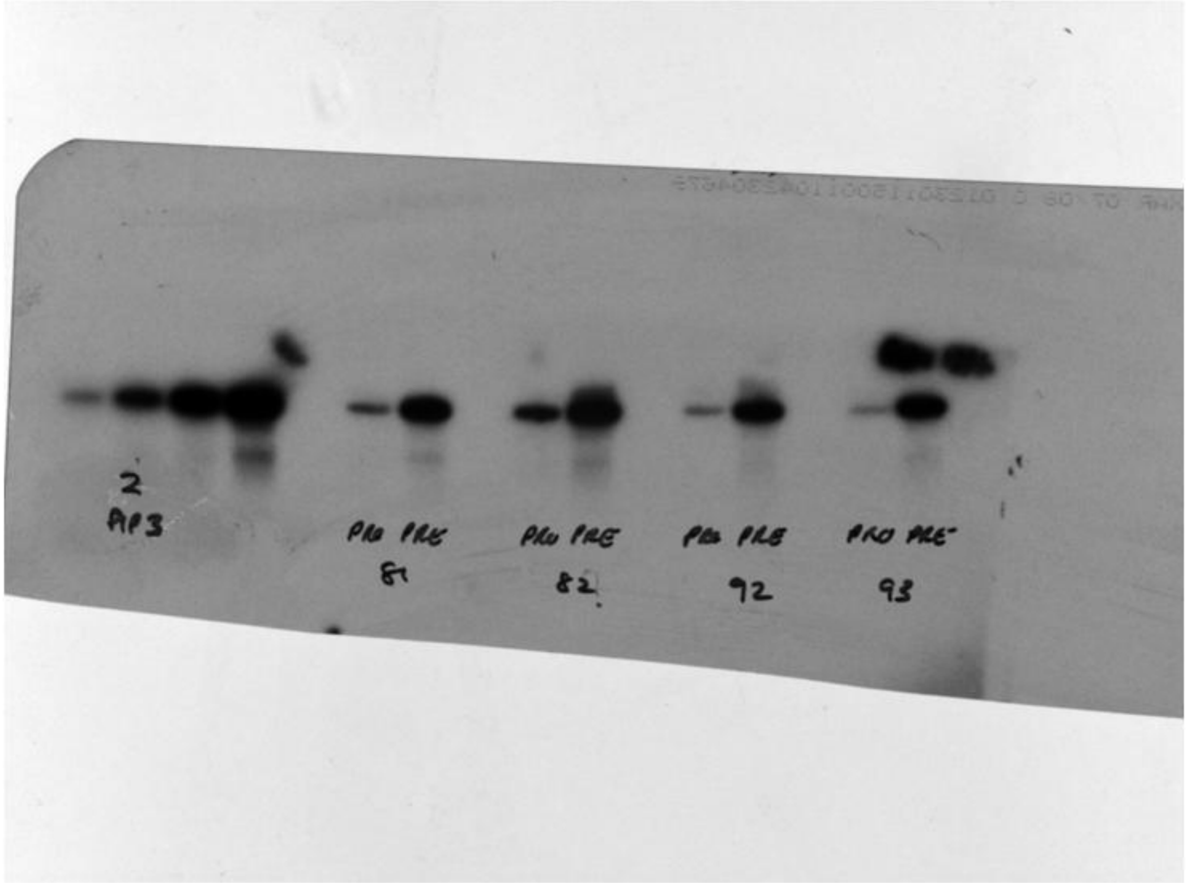

β-globin loading control for V-Jκ1 and Igλ recombination shown in Figures 2A and 2B

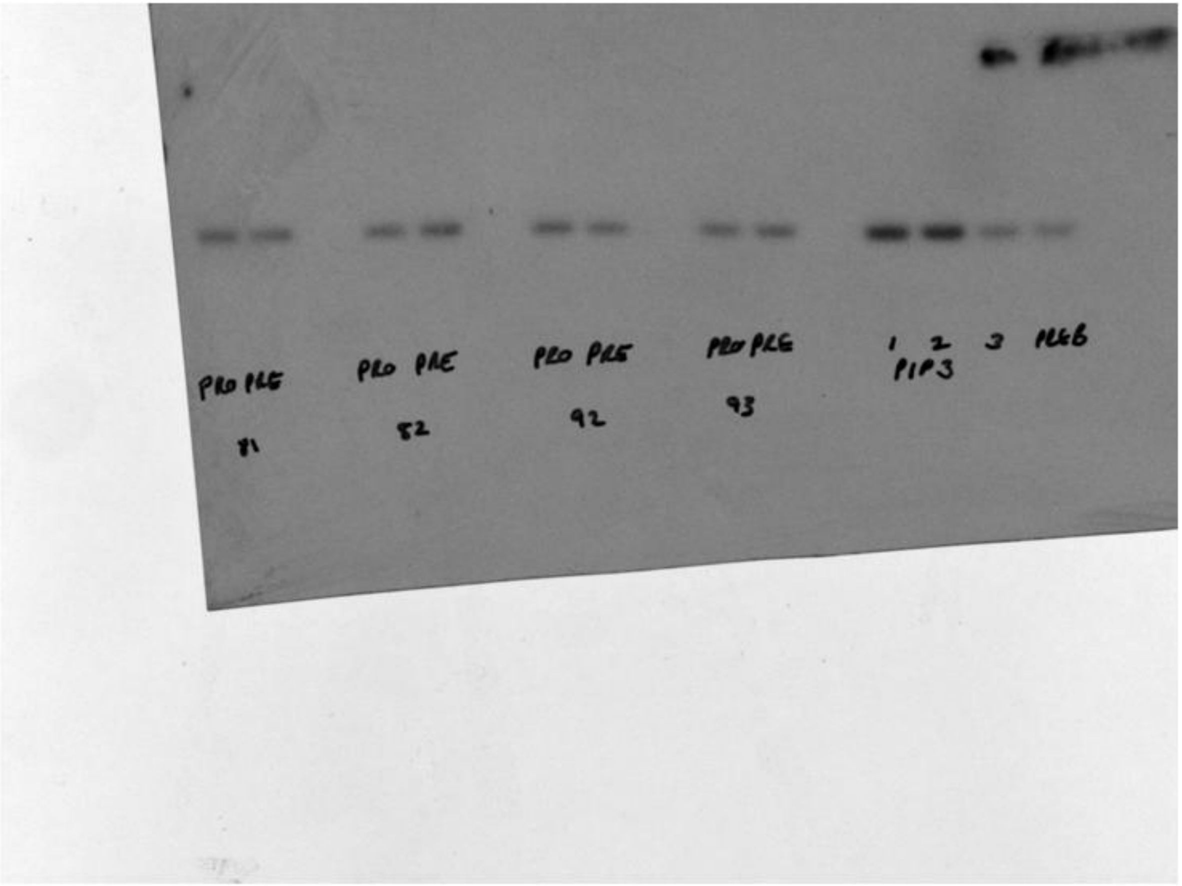

Supplement: Source data Fig. 2 [file emboj201342df2.pdf]
