# Supplementary material for: Transcription-coupled eviction of histones H2A/H2B governs V(D)J recombination
Source: EMBO J. 2013 Mar 5;32(10):1381–92. doi: 10.1038/emboj.2013.42 (PMC3655464; doi:10.1038/emboj.2013.42)

Explanation of source data

Figure 3A

For the *Hprt* loading controls, PCRs with two quantities of input DNA were performed to determine which gave the most similar signal between the non-transgenic and PIP transgenic samples. All *Hprt* control samples were run on the same blot; the non-transgenic samples on the lower part of the original blot were better normalised to the rest of the PIP samples on the upper part of this blot. Therefore these two lanes on the lower blot were substituted for those on the upper blot. The levels of sterile transcripts at κJC and Jλ1 were then performed with the normalised cDNA amounts shown in the Figure.

We also removed a lane showing the level of *Hprt* transcripts in non-transgenic pro-B cells that was originally run next to the pre-B cell sample since we felt this information was redundant.

Finally, we removed a lane showing the level of Jλ1 sterile transcripts in non-transgenic pro-B cells, originally run next to the pre-B cell sample since this information was also redundant.

We have marked the deleted lanes with asterisks and have added a sentence to the legend of Figure 3A to state: “The asterisks indicate where lanes have been removed from a continuous gel”.

Figures 3A and B, Hprt controls for κJC and Jλ1

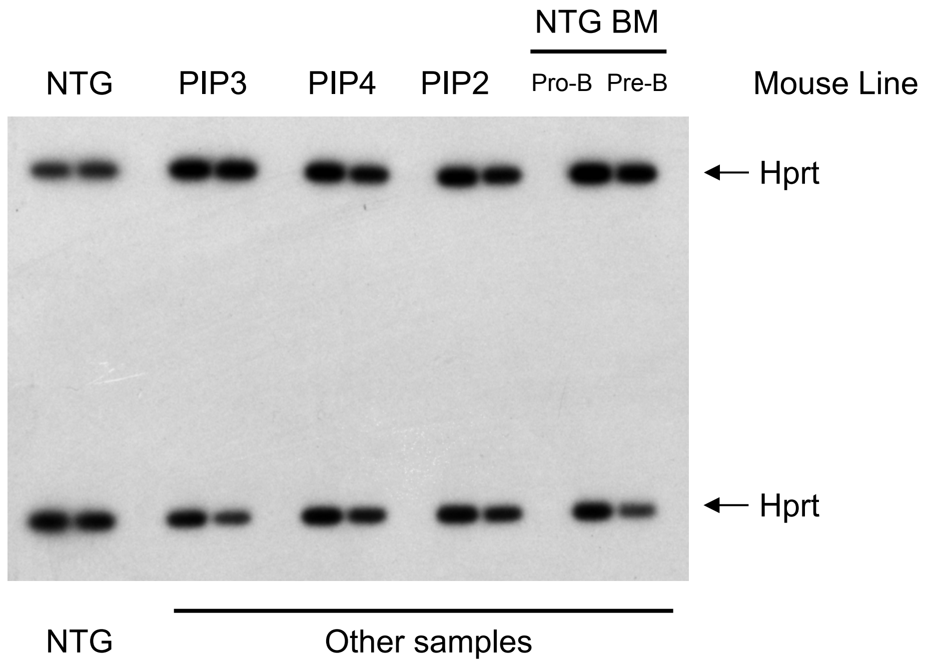

i) The NTG lanes, lower left, were substituted for the NTG lanes directly above.
ii) The pro-B NTG BM lane (top of gel, second from right) was deleted

Figure 3B

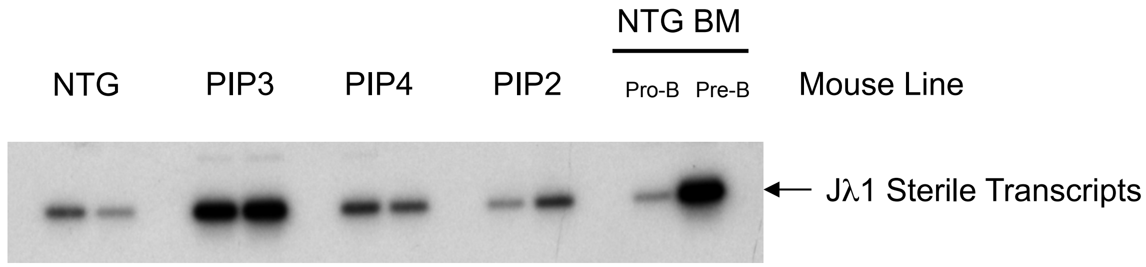

Jλ1 transcripts: The lane showing transcripts in NTG BM pro-B cells (second from right) was deleted to generate the final Figure.

HPRT controls

These data are shown in Figures 3A and 3B to normalise κJC and Jλ1 sterile transcripts

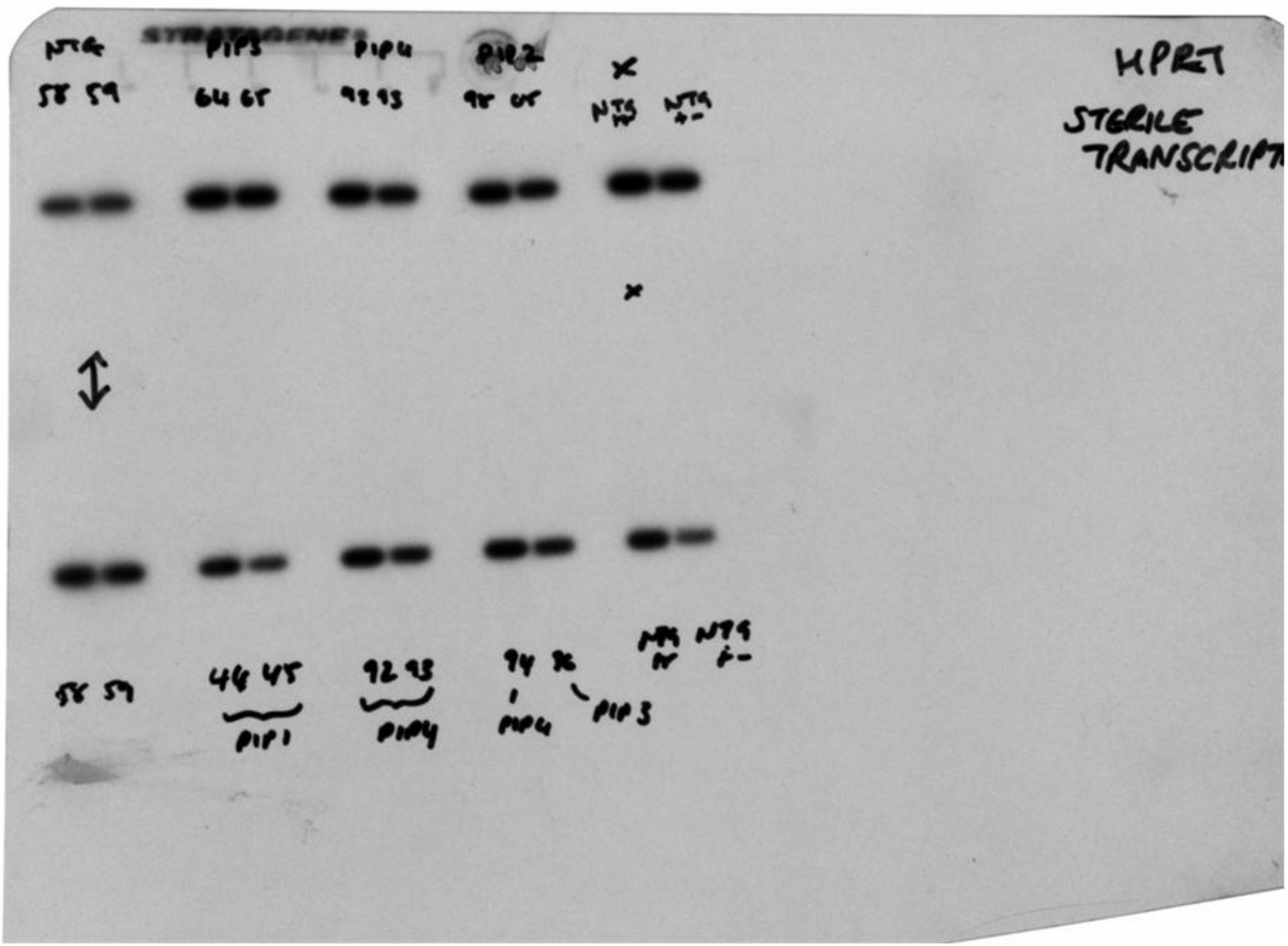

Jλ1 sterile transcription. These data are shown in Figure 3B

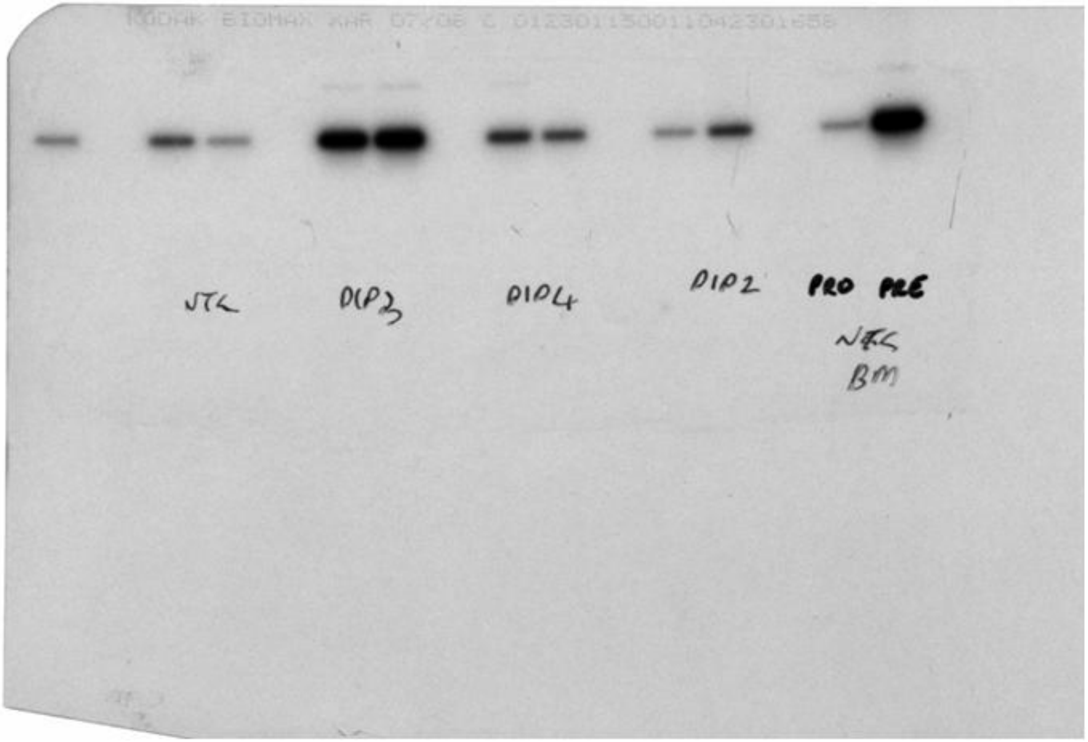

Supplement: Source data Fig. 3 [file emboj201342df3.pdf]
